# Attributable Cost of Adult Respiratory Syncytial Virus Illness Beyond the Acute Phase

**DOI:** 10.1093/ofid/ofae097

**Published:** 2024-02-22

**Authors:** Ahuva Averin, Mark Atwood, Reiko Sato, Kari Yacisin, Elizabeth Begier, Kimberly Shea, Daniel Curcio, Linnea Houde, Derek Weycker

**Affiliations:** Policy Analysis Inc, Boston, Massachusetts, USA; Policy Analysis Inc, Boston, Massachusetts, USA; Pfizer Inc, Collegeville, Pennsylvania, USA; Pfizer Inc, Collegeville, Pennsylvania, USA; Pfizer Inc, Collegeville, Pennsylvania, USA; Pfizer Inc, Collegeville, Pennsylvania, USA; Pfizer España, Madrid, Spain; Policy Analysis Inc, Boston, Massachusetts, USA; Policy Analysis Inc, Boston, Massachusetts, USA

**Keywords:** costs and cost analysis, respiratory syncytial virus, respiratory tract infections

## Abstract

**Background:**

Estimates of the cost of medically attended lower respiratory tract illness (LRTI) due to respiratory syncytial virus (RSV) in adults, especially beyond the acute phase, is limited. This study was undertaken to estimate the attributable costs of RSV-LRTI among US adults during, and up to 1 year after, the acute phase of illness.

**Methods:**

A retrospective observational matched-cohort design and a US healthcare claims repository (2016–2019) were employed. The study population comprised adults aged ≥18 years with RSV-LRTI requiring hospitalization (RSV-H), an emergency department visit (RSV-ED), or physician office/hospital outpatient visit (RSV-PO/HO), as well as matched comparison patients. All-cause healthcare expenditures were tallied during the acute phase of illness (RSV-H: from admission through 30 days postdischarge; ambulatory RSV: during the episode) and long-term phase (end of acute phase to end of following 1-year period).

**Results:**

The study population included 4526 matched pairs of RSV-LRTI and comparison patients (RSV-H: n = 970; RSV-ED: n = 590; RSV-PO/HO: n = 2966). Mean acute-phase expenditures were $42 179 for RSV-H (vs $5154 for comparison patients), $4409 for RSV-ED (vs $377), and $922 for RSV-PO/HO (vs $201). By the end of the 1-year follow-up period, mean expenditures—including acute and long-term phases—were $101 532 for RSV-H (vs $36 302), $48 701 for RSV-ED (vs $27 131), and $28 851 for RSV-PO/HO (vs $20 523); overall RSV-LRTI attributable expenditures thus totaled $65 230, $21 570, and $8327, respectively.

**Conclusions:**

The cost of RSV-LRTI requiring hospitalization or ambulatory care among US adults is substantial, and the economic impact of RSV-LTRI may extend well beyond the acute phase of illness.

## Teaser

Study findings indicate that acute-care costs for lower respiratory tract illness due to respiratory syncytial virus (RSV-LRTI) are high among US adults, and that attributable costs for RSV-LRTI remain high throughout the 1-year period following the acute phase of illness.

Respiratory syncytial virus (RSV) has long been recognized as a significant cause of acute respiratory illness in young children and is increasingly being documented as an important viral pathogen in older adults [1–4]. In many cases, RSV presents with symptoms like the common cold. However, adults with chronic medical conditions and elderly persons are at increased risk of severe illness from RSV (especially lower respiratory tract illness [LRTI]), which may lead to hospitalization and/or premature death [5].

While the economic burden of RSV among infants in the United States (US) has been relatively well documented in published literature, few studies have evaluated the economic burden of RSV among US adults [6–8]. In a 2022 study by Mesa-Frias et al, mean all-cause costs (2020 US$) among commercially insured US adults were higher by $975–$1208 (depending on data source) during the 1-week period before RSV diagnosis and $2679–$8173 during the 1-week period following RSV diagnosis, relative to the 2–8 weeks preceding the RSV diagnosis [6]. Weekly costs pre/post-RSV diagnosis increased with increasing age and were higher among younger adults with (vs without) comorbidities.

Costs beyond the acute phase were not considered in the Mesa-Frias et al study, nor were they considered in the 2 other US studies that employed older (pre-2014) data [6–8]. In the only published study to evaluate adult RSV costs during acute and long-term phases of illness, all-cause healthcare costs (2020 Canadian dollars) attributable to RSV among Canadian adults (laboratory-confirmed, mostly hospitalized/emergency department cases) were reported to range from $13 746 to $19 586 during the 30-day period following diagnosis and from $70 539 to $96 271 during the 365-day period following diagnosis [9]. Because of the limited data on the attributable cost of RSV beyond the acute phase of illness among US adults, and because of the limited data on the economic burden of RSV among US adults by age, comorbidity profile, and care setting, a new study was undertaken.

## METHODS

### Study Design and Data Source

A retrospective observational matched-cohort design and data from the Merative MarketScan Commercial Claims and Encounters (CCAE) and Medicare Supplemental and Coordination of Benefits (MDCR) Databases (the “MarketScan Database”) were employed. For this study, data from the MarketScan Database spanned January 2016 through December 2019.

The CCAE Database includes healthcare (ie, facility, professional-service, outpatient pharmacy) claims and enrollment information from employer-sponsored plans throughout the US that provide health benefits to >15 million working persons aged <65 years annually, including employees as well as their spouses and dependents. The MDCR database includes healthcare claims and enrollment information for retirees who are Medicare eligible and have elected to enroll in an employer-sponsored Medicare supplemental plan and for which both the Medicare-paid amounts and employer-paid amounts are available.

Data available for each facility and professional-service claim include the dates and places of service, diagnoses, procedures performed/services rendered, and quantity of services (professional-service claims). Data available for each outpatient pharmacy claim include the drug dispensed, dispensing date, dose, quantity dispensed, and number of therapy days supplied. Medical and pharmacy claims also include amounts paid (ie, reimbursed) by health plans and patients to healthcare providers for services rendered. Selected demographic and eligibility information is also available. Patient-level data can be arrayed chronologically to provide a detailed longitudinal profile of all medical and pharmacy services used by each plan member.

### Study Population

#### RSV-LRTI Patients

Beginning in January 2017, and continuing monthly thereafter through December 2018, all adults aged ≥18 years in the source population who had evidence of a medically attended episode of LRTI due to RSV (RSV-LRTI) were identified. Episodes were ascertained based on hospitalizations with a diagnosis code for RSV-LRTI in the principal position, RSV in the principal position and LRTI in the secondary position, or LRTI in the principal position and RSV in the secondary position; and ambulatory encounters with a diagnosis code(s)—in any position—indicating RSV and LRTI on the same date or within ±10 days (Supplementary Appendix A).

All encounters with a diagnosis code for LRTI occurring within 10 days before first evidence of RSV, and all encounters with a diagnosis code for LRTI or RSV occurring within 30 days after first evidence of RSV or LRTI (whichever occurred first), were considered part of the same episode (ie, the maximum duration of an episode was assumed to be 30 days). Episodes including a hospitalization for RSV-LRTI (irrespective of ambulatory RSV-LRTI encounters during the episode) were considered hospitalized episodes (RSV-H). Episodes not including a hospitalization for RSV-LRTI were considered ambulatory episodes and were stratified based on care setting (ie, using a hierarchical approach: emergency department [RSV-ED], if not, then physician office/hospital outpatient [RSV-PO/HO]).

Patients who experienced >1 episode during the study period contributed only their first unique episode to the analysis. Patients without comprehensive health benefits during the 1-year period preceding the beginning of the episode, those with evidence of RSV during the 90-day period preceding the beginning of the episode, and patients with missing data on healthcare expenditures were excluded from the study population.

#### Comparison Patients

Each RSV-LRTI patient was matched to 1 “comparison patient” from the source population in the same month. Matching was implemented for each RSV-LRTI patient by first identifying all “candidate” comparison patients who, in the match month and during their history period prior to the match month, had comprehensive health benefits and the same chronological single year of age, sex, insurance type, and comorbidity profile, and who did not have any evidence of RSV-LRTI during the study period. Care setting was not considered in the matching process. From all such comparison candidates, 1 was randomly selected for matching, and for each matched pair, the beginning of the RSV-LRTI episode was designated as their “index date.” Once matched, both the RSV-LRTI patient and comparison patient were included in the study population and removed from the source population. The same process was repeated for each subsequent calendar month, using the pool of patients remaining in the source population after matching in prior months.

### Study Outcomes

All-cause healthcare expenditures for RSV-LRTI patients and comparison patients were tallied during the acute illness phase as well as the long-term phase. For hospitalized RSV-LRTI patients, the acute phase included the index RSV hospitalization and the 30-day postdischarge period; the long-term phase included the 1-year period following the end of the 30-day postdischarge period. For ambulatory RSV-LRTI patients, the acute phase included the index episode, and the long-term phase included the subsequent 1-year period. All-cause healthcare expenditures among comparison patients were evaluated during the corresponding periods. Expenditures were tallied using paid amounts on corresponding healthcare claims and were expressed in 2020 US dollars using the appropriate component (eg, hospital and related services) of the Consumer Price Index for All Urban Consumers [10].

All-cause healthcare expenditures during RSV-LRTI episodes were also tallied using a disease-attributable approach. With this approach, disease-related services during the episode were identified based on encounters with diagnosis codes for RSV and/or LRTI; hospitalizations were limited to those with a corresponding principal diagnosis code.

### Baseline Characteristics

Baseline characteristics included age, sex, insurance type, and comorbidity profile. Comorbidity profile was defined based on the presence or absence of chronic medical conditions (CMCs) listed in the Centers for Disease Control and Prevention US Advisory Committee on Immunization Practices recommendations for influenza vaccination, and was classified as follows: CMC^–^, immunocompetent without CMC; CMC^+^, immunocompetent with CMC; and IC, immunocompromised [11]. Presence of medical conditions was ascertained during the 1-year period preceding the beginning of the RSV-LRTI episode and was identified based on ≥2 outpatient diagnoses (≥7 days apart) or ≥1 inpatient diagnosis (Supplementary Appendix B and Supplementary Appendix C).

### Statistical Methods

Characteristics of RSV-LRTI patients and comparison patients were summarized using descriptive statistics (ie, mean with standard deviation [SD], percentages). Healthcare expenditures attributable to RSV-LRTI—by care setting—were calculated based on the difference in all-cause expenditures between RSV-LRTI patients and comparison patients and were analyzed on an overall basis as well as by age and comorbidity profile. Expenditures during the long-term phase were adjusted for differential follow-up using a population-based approach. Because study measures were not normally distributed, techniques of nonparametric bootstrapping were employed to characterize 95% confidence intervals (CIs) for study measures [12]. With this technique, repeated samples (1000 in total, with replacement) were drawn from the study population and mean expenditures were calculated for each sample; 95% CIs were then constructed based on the empirical distribution of the sample-specific means. Analyses were conducted using SAS version 9.4 software.

## RESULTS

### Population Characteristics

A total of 6554 patients had an episode of RSV-LRTI (22% hospitalized, 78% ambulatory) between January 2017 and December 2018 (Table 1). Among these patients, 77% (n = 5032) met all remaining eligibility criteria, and among these patients, 90% (n = 4526) were matched to comparison patients and thus included in the study population. Mean age of the study population was 56 (SD, 18) years and 61% were female (Table 2). Nearly one-half (47%) of RSV-LRTI patients (and thus comparison patients) were classified as CMC^–^, 43% as CMC^+^, and 10% as IC.

**Table 1.** Selection of Study Population

Selection Criteria	No. of Patients
RSV-LRTI patients	
Inclusion criteria	
RSV-LRTI episode between January 2017 and December 2018	6554
Hospitalization for RSV-LRTI	1462
Ambulatory encounter for RSV-LRTI	5092
Exclusion criteria	
<12 mo of healthcare coverage prior to RSV-LRTI episode	5156
Evidence of RSV during 90-d period preceding beginning of RSV-LRTI episode	5032
Matched pairs of RSV-LRTI patients and comparison patients	4526

Abbreviation: RSV-LRTI, lower respiratory tract illness due to respiratory syncytial virus.

**Table 2.** Baseline Characteristics of Patients With Lower Respiratory Tract Illness due to Respiratory Syncytial Virus and Matched Comparison Patients*

Characteristic	RSV-LRTI and Comparison Patients
	(N = 4526 Pairs)
Age, y, mean (SD)	55.6 (18.0)
Age group, y	
18–49	34.1%
50–64	39.3%
65–74	11.0%
75–84	8.9%
≥85	6.7%
Sex	
Female	60.8%
Male	39.2%
Insurance type	
Commercial fee-for-service	64.7%
Commercial encounter	8.4%
Medicare fee-for-service	22.6%
Medicare encounter	4.4%
Comorbidity profile	
CMC^–^	46.9%
CMC^+^	43.1%
Chronic cardiopulmonary	0.1%
Chronic cardiovascular	16.5%
Chronic hematologic	4.9%
Chronic hepatic	0.6%
Chronic metabolic	17.4%
Chronic neurologic	5.8%
Chronic pulmonary	21.9%
Chronic renal	3.2%
Obesity (BMI >40 kg/m^2^)	3.6%
Immunocompromised	10.0%

Abbreviations: BMI, body mass index; CMC^–^ immunocompetent without chronic medical conditions; CMC^+^, immunocompetent with chronic medical conditions; RSV-LRTI, lower respiratory tract illness due to respiratory syncytial virus; SD, standard deviation.

^*^Baseline characteristics ascertained during 12-month history period.

For hospitalized episodes, mean (95% CI) all-cause healthcare expenditures during the acute phase of illness were $42 179 ($38 179–$46 194) for RSV-LRTI patients, versus $5154 ($3984–$6605) for comparison patients, corresponding to attributable expenditures totaling $37 025 ($33 922–$40 068) (Table 3). For ambulatory episodes, mean (95% CI) acute phase expenditures were $4409 ($3975–$4885) for RSV-ED patients and $922 ($796–$1059) for RSV-PO/HO patients; corresponding values for comparison patients were $377 ($186–$598) and $201 ($134–$281), and thus attributable expenditures totaled $4032 ($3696–$4396) and $721 ($612–$832), respectively. By the end of the 1-year follow-up period, mean (95% CI) all-cause expenditures (acute plus long-term phases) were $101 532 ($90 689–$112 541) for RSV-H patients (vs $36 302 [$32 154–$41 060]), $48 701 ($32 253–$76 187) for RSV-ED patients (vs $27 131 [$18 505–$38 185]), and $28 851 ($25 110–$32 548) for RSV-PO/HO patients (vs $20 523 [$17 980–$23 222]); overall RSV-attributable expenditures thus totaled $65 230 ($57 167–$73 404), $21 570 ($4811–$42 337), and $8327 ($5064–$11 535), respectively.

**Table 3.** All-Cause Healthcare Expenditures (Mean [95% Confidence Interval]) During 1-Year Period From Beginning of Lower Respiratory Tract Illness due to Respiratory Syncytial Virus Episode

Type of Episode	RSV-LRTI Patients	Comparison Patients	Difference
	(N = 4526)	(N = 4526)	
Hospitalized RSV-LRTI	(n = 970)	(n = 970)	
Acute phase	42 179 (38 179–46 194)	5154 (3984–6605)	37 025 (33 922–40 068)
Index hospitalization	31 626 (28 789–35 074)	1289 (686–2276)	30 338 (27 942–32 871)
End of index hospitalization through end of 30-d follow-up	10 553 (8590–12 802)	3865 (2994–4887)	6688 (5065–8451)
Long-term phase			
End of acute phase through end of 1-y follow-up	59 353 (50 561–68 734)	31 148 (27 354–35 294)	28 205 (21 898–34 906)
Total	101 532 (90 689–112 541)	36 302 (32 154–41 060)	65 230 (57 167–73 404)
Ambulatory RSV-LRTI			
Emergency department	(n = 590)	(n = 590)	
Acute phase			
Index ambulatory encounter	4409 (3975–4885)	377 (186–598)	4032 (3696–4396)
Long-term phase			
End of acute phase through end of 1-y follow-up	44 292 (27 789–71 515)	26 754 (18 084–37 803)	17 538 (640–38 534)
Total	48 701 (32 253–76 187)	27 131 (18 505–38 185)	21 570 (4811–42 337)
Physician office/hospital outpatient	(n = 2966)	(n = 2966)	
Acute phase			
Index ambulatory encounter	922 (796–1059)	201 (134–281)	721 (612–832)
Long-term phase			
End of acute phase through end of 1-y follow-up	27 928 (24 235–31 534)	20 322 (17 794–23 002)	7606 (4362–10 841)
Total	28 851 (25 110–32 548)	20 523 (17 980–23 222)	8327 (5064–11 535)

Data are presented as mean (95% confidence interval) US dollars.

Abbreviation: RSV-LRTI, lower respiratory tract illness due to respiratory syncytial virus.

Mean (95% CI) acute phase and long-term phase attributable expenditures for RSV-LRTI patients aged 18–64 years were $78 820 ($66 559–$90 897) for RSV-H, $23 111 ($3336–$50 394) for RSV-PO/HO, and $5738 ($3095–$8526) for RSV-ED (Supplementary Table 1); for patients aged ≥65 years, corresponding expenditures were $50 037 ($41 441–$59 764), $16 882 ($−4571 to $35 187), and $18 516 ($7758–$30 420), respectively. In general, attributable expenditures were higher among adults with comorbidities (CMC^+^ or IC) than adults without comorbidities across all care settings (Supplementary Table 2). Costs of RSV-LRTI episodes that were tallied using a disease-attributable approach were comparable to acute-phase attributable costs described above (ie, those based on differences between RSV-LRTI patients and comparison patients) (Supplementary Table 3).

## DISCUSSION

In this retrospective observational study using a large healthcare claims database, we evaluated the attributable costs of RSV-LRTI—by care setting—during the acute phase of illness, as well as beyond the acute phase, among commercially insured US adults aged ≥18 years on an overall basis and within subgroups defined on age and comorbidity profile. Findings from analyses indicate that the attributable cost of RSV-LRTI during the acute phase of illness may be higher than values reported in previously published studies, and that the clinical and economic consequences of RSV-LRTI may extend well beyond the acute phase of illness. Study findings also indicate that the attributable costs of RSV-LRTI during both the acute and long-term phases are notably higher among adults with chronic medical conditions or immunocompromising conditions relative to immunocompetent adults without chronic medical conditions.

Our study is the first to report acute phase and long-term attributable costs of RSV-LRTI among US adults by care setting, and thus—due to differences in study designs—results described herein are not directly comparable with previous research. We do note, however, that a weighted average of acute phase RSV-LRTI costs across care settings in our study yielded a higher estimate (∼$9000) than that reported by Mesa-Frias and colleagues ($4000–$9000, depending on age and data source), presumably due—at least in part—to our use of a 30-day window for defining acute episodes versus the 1 week before/after RSV diagnosis employed in the Mesa-Frias study [6]. We also note that a weighted average of long-term RSV-LRTI costs across care settings in our study yielded a higher estimate than that reported in the study by Amand and colleagues ($22 000 vs $13 000 [adjusted to 2020 US dollars]) [7]. Finally, we note that trends between acute phase and long-term RSV-LRTI costs were comparable between our study and the Canadian study by Rafferty et al [9].

Although healthcare claims databases provide information on large numbers of patients with specific diagnoses who receive care for specific conditions, several limitations from the use of such databases should be noted. While algorithms for identifying RSV-LRTI encounters (ie, based on diagnosis codes) have been used in prior studies focusing on infant and adult populations, they have not been formally evaluated against a “gold standard” within the study data sources and thus their accuracy (ie, in terms of sensitivity and specificity) is unknown. In addition, our study employed a maximum 30-day interval for defining RSV-LRTI episodes based on clinical guidance indicating that, while infections should resolve within 14 days for most patients, some with more severe disease may have additional healthcare encounters beyond the 14-day interval that are appropriately attributable to the index episode. While use of a longer maximum episode duration may yield higher estimated acute-phase costs, we note that few episodes spanned >14 days (RSV-H: 7%, RSV-ED: 2.5%, RSV-PO/HO: 4.8%). Moreover, use of a matched-cohort design to estimate disease-attributable costs should minimize the potential for bias due to the possible inclusion of non-RSV-related expenditures. Although most adult patients with LRTI are not tested for specific pathogens (such as RSV) in clinical practice, and pathogens detected via testing are not always recorded in healthcare claims, we note that findings from a recent validation study suggest that algorithms for identifying RSV hospitalizations based on *International Classification of Diseases, Tenth Revision* diagnosis codes are reasonably sensitive and highly specific [13].

Because RSV-LRTI episodes in this study may not be representative of all patients with RSV-LRTI, attributable costs of RSV-LRTI may be misestimated. Use of operational algorithms to characterize comorbidity profiles, which have been employed in several previously published studies, undoubtedly resulted in misclassification of some adults who have the underlying conditions as well as some adults who do not have the underlying conditions [14–18]. Use of drugs (ie, those obtained via outpatient pharmacy) was not considered in tallying costs, and thus study findings may be downwardly biased somewhat. The true cost of healthcare is not available in the study data sources, and thus expenditures (ie, amounts paid by health plans and patients for services rendered) were employed. Finally, adults without health insurance are not represented in the study data sources; caution should be used when generalizing study results to other populations and settings.

In conclusion, the cost of RSV-LRTI requiring hospitalization or ambulatory care among US adults is substantial, and the economic impact of RSV-LTRI may extend well beyond the acute phase of illness. Strategies to prevent RSV-LRTI among adults—including the recent recommendation for RSV vaccination among US adults aged ≥60 years—have the potential to yield considerable economic benefits to the US healthcare system [19].

## Supplementary Material

ofae097_Supplementary_Data

## Supplementary Data

Supplementary materials are available at *Open Forum Infectious Diseases* online. Consisting of data provided by the authors to benefit the reader, the posted materials are not copyedited and are the sole responsibility of the authors, so questions or comments should be addressed to the corresponding author.

## Notes

***Author contributions.*** Conception and design: All authors. Acquisition of data: K. S. and D. W. Analysis or interpretation of data: All authors. Preparation of the manuscript: A. A., R. S., and D. W. Critical review of the manuscript: M. A., L. H., K. Y., E. B., K. S., and D. C. All authors have read and approved the final version of the manuscript.

***Data availability.*** Data were provided under a data use agreement and therefore are not publicly available.

***Patient consent.*** This study does not include factors necessitating patient consent.

***Disclaimer.*** The study sponsor, Pfizer Inc, reviewed the study research plan and study manuscript; data management, processing, and analyses were conducted by Policy Analysis Inc. All final analytic decisions and the decision to submit for publication were made solely by study investigators.

***Financial support*.** This work was supported by Pfizer Inc.
